# Diagnostic value of microRNA panel in endometrial cancer: A systematic review

**DOI:** 10.18632/oncotarget.27601

**Published:** 2020-05-26

**Authors:** Hannah Donkers, Ruud Bekkers, Khadra Galaal

**Affiliations:** ^1^Royal Cornwall Hospital NHS Trust, Truro, Cornwall, United Kingdom; ^2^Grow School for Oncology and Developmental Biology, Maastricht University, The Netherlands; ^3^Catharina Hospital, Eindhoven, The Netherlands; ^4^Exeter University, Exeter, United Kingdom

**Keywords:** diagnostic tests, diagnostic biomarkers, endometrial cancer, MicroRNAs, uterine neoplasms

## Abstract

Purpose: We conducted a systematic review to evaluate the overall diagnostic accuracy of miRNAs in detecting endometrial cancer.

Materials and Methods: A systematic search of Medline, Embase, Cinahl and the Cochrane Controlled Register of Trials was performed to identify studies reporting on the diagnostic value of miRNA in EC patients. Included were diagnostic studies looking at miRNA expression in women diagnosed with endometrial cancer. Two reviewers independently selected studies and assessed quality of studies using the Quality Assessment of Diagnostic Accuracy Studies 2 (QUADAS-2) score system. Data extraction was completed and the vote-counting strategy was used to rank miRNAs.

Results: 26 studies were included with a total number of 1,400 EC patients reporting on 106 differentially expressed miRNAs. The most frequently found up-regulated miRNA was miR-205 followed by miR-200c, -223, -182, -183 and -200a. In addition, miR-135b, miR-429, miR-141 and miR-200b were also frequently up-regulated. There was less consensus on down-regulated miRNAs.

Conclusions: miRNAs yield a promising diagnostic biomarker potential in endometrial cancer, especially miR-205, the miR-200 family and miR-135b, -182, -183 and -223. However, no sufficient high quality data are available to draw hard conclusions. More research is needed to validate the diagnostic potential of these miRNAs in larger studies. In addition, the potential of urine as a non-invasive biofluid should be investigated in more detail.

## INTRODUCTION

Endometrial cancer (EC) is the most common malignancy of the female genital tract and the 8th cause of death in women in the United Kingdom (UK) [1]. Two different subtypes of EC have been described: type I tumours are mostly endometrioid adenocarcinomas and are associated with unopposed oestrogen stimulation and obesity and are often preceded by endometrial hyperplasia [2]. Type II tumours on the other hand are predominantly serous carcinomas, are commonly described as oestrogen independent arising in atrophic endometrium and are less well differentiated and therefore haver poorer prognosis [2, 3]. The large majority of endometrial cancer are type I endometrioid, which is associated with good prognosis [4]. This is largely because women present early with bleeding problems and are therefore diagnosed at an early stage [5]. However, between 15 to 25% of women present with advanced stage disease (stage III or stage IV) with a 5-year survival varying from 40% to 79% for FIGO stage III, and from 0% to 24% for FIGO stage IV disease [6].

At the moment, diagnosis of EC is made by combination of transvaginal ultrasound scan (TVUS) and endometrial biopsy, which is an invasive and uncomfortable investigation with Visual Analogue Scale (VAS) pain score of 6.5 in postmenopausal women [7, 8]. In addition, high numbers of technical problems (12–23%) and insufficient amount of tissue (16–68%) in obtaining endometrial biopsy have been described [9]. Therefore, the identification of validated and non-invasive diagnostic biomarkers are needed. These biomarkers need to be accurate in order to improve earlier diagnosis and outcomes including survival.

MicroRNAs (miRNAs) are small noncoding RNAs involved in posttranscriptional regulations of various cellular processes and over 2,000 human miRNAs are identified [10, 11]. MiRNAs have been demonstrated to play a major role in a wide range of developmental processes including metabolism, cell proliferation, apoptosis and developmental timing [12]. Overexpressed miRNAs may function as both oncogenes (through downregulation of tumour-suppressor genes) and/or regulator of cellular processes such as cell differentiation or apoptosis. This is thought to be how miRNAs are associated with the development of different cancer types such as colorectal, breast, ovarian and endometrial cancer [13–16], although the exact pathways are not entirely understood. Because of their potential role as agents controlling cell growth and differentiation, miRNAs have been proposed to be good candidates for cancer diagnosis and therapy [17]. In addition, previous systematic reviews show a promising diagnostic potential of miRNA in cancer types such as ovarian and pancreatic cancer [18, 19], however the diagnostic value of miRNA for endometrial cancer remains unclear. Results of published studies are inconsistent due to differences in study design, specimen types and miRNAs and different groups have obtained conflicting conclusions.

MiRNAs can be detected in fixed tissue specimens but also in blood, serum, urine and other body fluids [20]. To date it is unclear which specimen type can be used to achieve the most reliable and feasible biomarker for the detection of EC.

Therefore, this systematic review was conducted to summarize the global research and to evaluate the potential diagnostic value of miRNAs in detecting endometrial cancer. The aim of this systematic review is to provide guidance for future researchers as to which aspects of miRNA expression in EC warrants further exploration.

## RESULTS

### Study selection

**Figure 1:**
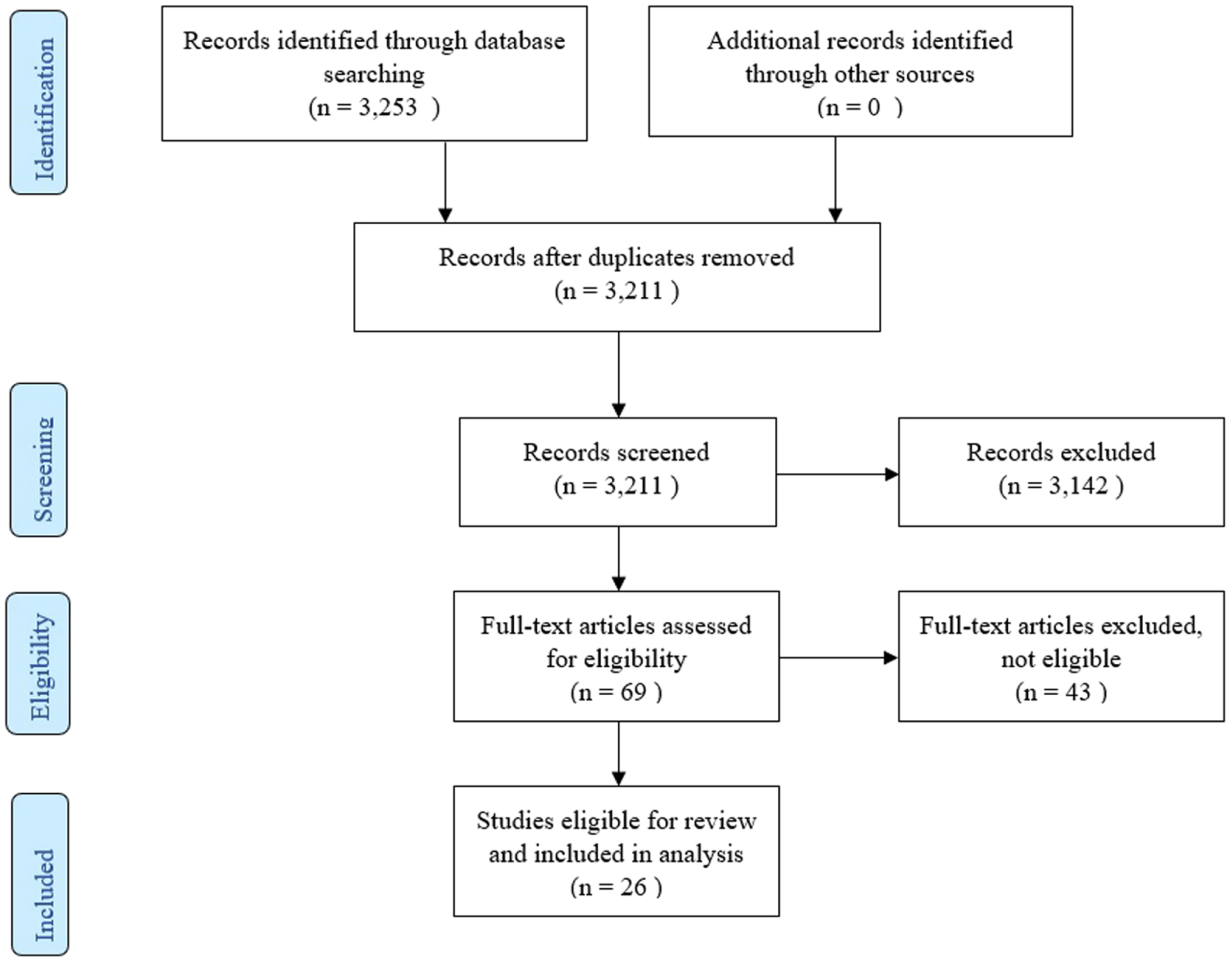
PRISMA flow diagram.

The flow diagram of the selected studies is depicted in Figure 1. The initial literature search identified 3,253 articles, from which 42 duplicates were excluded. Of the remaining 3,211 articles, 3,142 were excluded based on title and abstract screening. The search identified 69 full texts, of which 43 articles were excluded for the following reasons:

*In vitro* studies (using cell lines)

Not diagnostic

Only included patients after adjuvant radiotherapy

Compared endometrial cancer with ovarian cancer

Did not have a comparison group

Abstracts only

Only focused on sarcomas

Focused on the prediction of lymph node metastasis and miRNA expression

In addition, no other potential articles from the references of other reviews in the full-text screening process were found. Finally, 26 articles were included in this systematic review.

### Study characteristics and quality assessment

**Figure 2:**
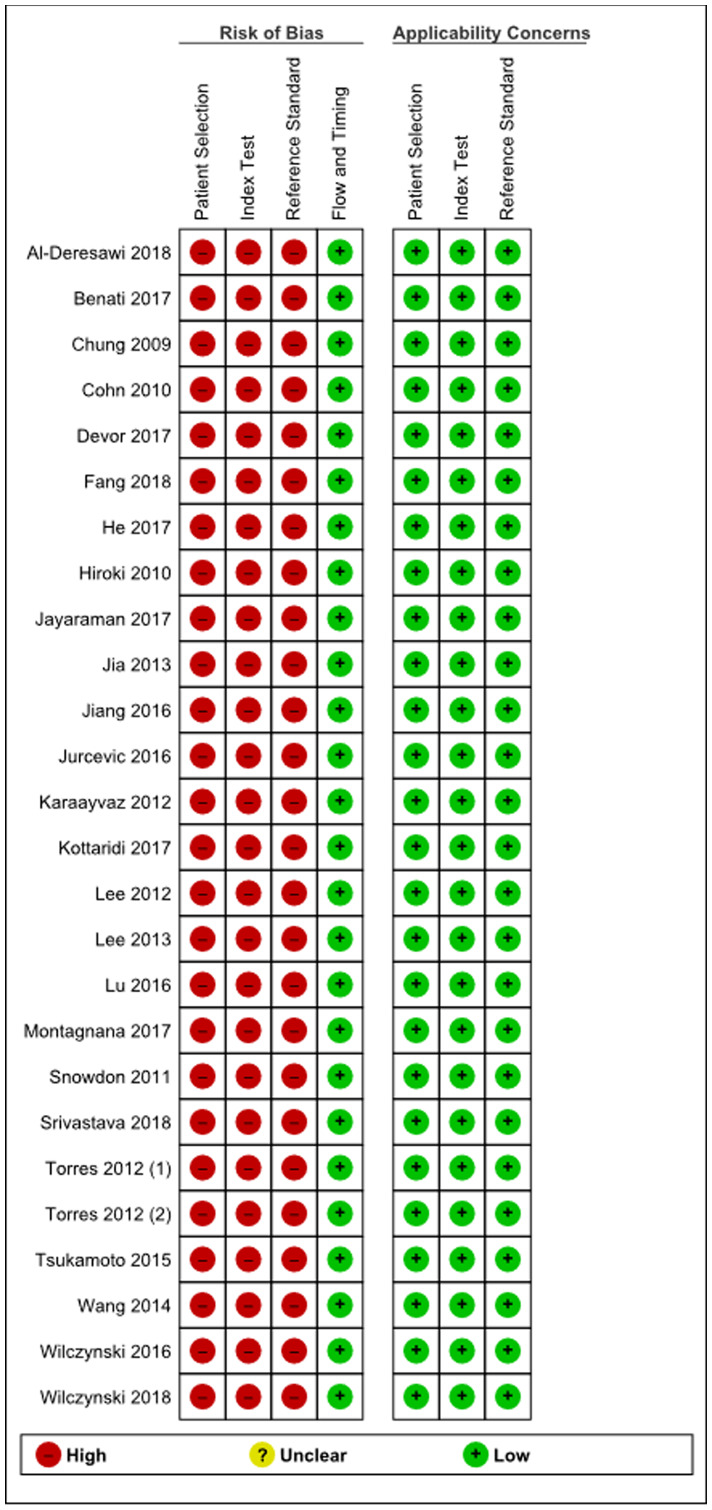
Summary of bias risk assessment results for QUADAS-2.

**Figure 3:**
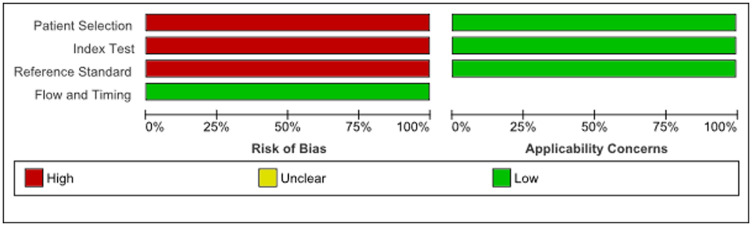
Quality of included studies according to QUADAS-2 guidelines.

The principal characteristics of the included studies are outlined in Supplementary Table 1. In this review, 26 articles were included with a total of 2,110 women of which 1,400 had endometrial cancer, 71 had either simple or complex atypical hyperplasia and 639 women had benign endometrium or polyps. The majority of studies were conducted in Asia (12 articles), 8 in Europe and 6 articles were conducted in United States/Canada. There were 16 studies that detected miRNAs in tissue specimens (Formalin-Fixed, Paraffin-Embedded (FFPE) or fresh frozen tissue), 5 studies that used serum [15, 21–24], 1 study detected miRNA in plasma [25] and 1 study used urine as bio-fluid [26]. Two studies used both tissue and plasma samples and one study used both liquid based cytology (LBC) endometrial samples and tissue samples [27]. The majority of the studies only included endometrioid endometrial adenocarcinomas (13 articles), 6 did not specify which subtype they included and 5 articles included all subtypes of EC (type I and II) [15, 28–31]. Furthermore 1 article only included serous endometrial carcinomas [32] and 1 article included both serous endometrial carcinomas and endometrioid endometrial carcinomas but no other EC subtypes [33]. All studies used real-time quantitative reverse transcription polymerase chain reaction (qRT-PCR) methods to detect miRNA expression, either solely or after microarray or Northern Blot analysis. The risk of bias and applicability of these studies were evaluated based on QUADAS-2 and summarized in Figures 2 and 3. There was a high risk of bias in all studies on patient selection, index test and reference standard but a low risk of bias for flow and timing. Furthermore, there were no applicability concerns in any of the studies included in this review.

### Differentially expressed miRNAs from ranking

A total of 106 differentially expressed miRNAs were identified of which 19 were reported in at least two studies. Out of these 106 miRNAs, 55 were upregulated (Table 1). The most frequently found up-regulated miRNA was miR-205, which was reported in 10 articles and showed a mean fold change of 198.08 when tested in 134 EC patients and 64 control patients. Furthermore miR-200c was reported in 8 articles to be up-regulated in EC (mean fold change 27.99), in addition the following miRNAs were reported in 5 articles: miR-223 (mean fold change 40.17), miR-182 (mean fold change 11.41), miR-183 (mean fold change 8.75) and miR-200a (mean fold change 5.20).

**Table 1:** Up-regulated miRNAs reported in all studies in all specimen types with fold changes

MiRNA	Number of studies	Sample size EC	Sample size control	Mean fold change^**^	Studies with fold change reported
miR-205	10	134	64	198.08	4
miR-200c	8	164	83	27.99	4
miR-223	5	144	99	40.17	3
miR-182	5	141	79	11.41	3
miR-183	5	141	79	8.75	2
miR-200a	5	155	89	5.20	4
miR-135b	3	96	35	35.59	2
miR-429	3	117	61	6.83	3
miR-141	3	103	51	4.25	2
miR-200b	3	103	51	3.86	2
miR-200a^*^	2	73	31	26.96	1
miR-222	2	26	22	19.16	1
miR-141-3p	2	77	68	15.36	2
miR-200c-3p	2	28	62	15.34	1
miR-186	2	26	22	11.39	1
miR-200b^*^	2	73	31	6.52	1
miR-15b	2	40	49	6.10	1
miR-106a	2	68	48	2.80	2
miR-135a	1	23	4	34.05	1
miR-205-5p	1	28	62	24.19	1
miR-182-5p	1	28	62	22.76	1
miR-200b-3p	1	28	62	16.19	1
miR-92a	1	73	31	15.63	1
miR-9-5p	1	16	34	15.05	1
miR-27a	1	40	49	5.63	1
miR-210	1	38	28	5.23	1
miR-96	1	73	31	4.27	1
miR-194	1	38	28	4.11	1
miR-95	1	38	28	4.09	1
miR-155	1	38	28	3.87	1
miR-18a^*^	1	30	20	3.65	1
miR-222-3p	1	12	28	3.43	1
miR-96-5p	1	49	6	3.20	1
miR-103	1	38	28	3.00	1
miR-151	1	38	28	2.85	1
miR-34a	1	30	20	2.63	1
miR-92a-1^*^	1	30	20	2.47	1
miR-887-5p	1	50	50	2.41	1
miR-20a^*^	1	30	20	2.34	1
miR-106b^*^	1	30	20	2.34	1
miR-449a	1	73	31	2.26	1
miR-17^*^	1	30	20	1.99	1
miR-185	1	30	20	1.85	1
miR-1228	1	73	31	1.18	1
miR-146	1	141	20	NR	0
miR-425	1	141	20	NR	0
miR-1290	1	73	31	NR	0
miR-944	1	68	20	NR	0
miR-16	1	67	15	NR	0
miR-128a	1	67	15	NR	0
miR-148b	1	67	15	NR	0
miR-196a	1	67	15	NR	0
miR-301	1	67	15	NR	0
miR-582-5p	1	67	15	NR	0
miR-499	1	28	14	NR	0

NR = not reported, ^**^mean fold change as found by qRT-PCR.

Other promising up-regulated miRNAs listed in 3 articles were: miR-135b, miR-429, miR-141 and miR-200b. MiRNAs only listed up-regulated in one or two articles can be found in Table 1.

There was less consensus on miRNAs that are down-regulated in women with endometrial cancer (Table 2), with 44 different miRNAs being down-regulated. However, all miRNAs were only reported in 1 article [15, 21, 30, 32, 34–39]. MiR-137 and miR-129-3p showed the largest mean fold change (115.15 and 42.30 respectively) but were only found in a small cohort (sample size EC 23 and sample size control 4 patients) [33]. MiR-410 was found to have a mean fold change of 13.91 and was tested in a slightly larger cohort (sample size EC 73 and sample size control 31 patients) [35].

**Table 2:** Down-regulated miRNAs reported in all studies in all specimen types with fold changes

MiRNA	Number of studies	Sample size EC	Sample size control	Mean fold change^**^	Studies with fold change reported
miR-137	1	23	4	115.15	1
miR-129-3p	1	23	4	42.30	1
miR-410	1	73	31	13.91	1
miR-503	1	14	10	8.60	1
miR-1247	1	30	20	5.31	1
miR-376c	1	30	20	3.64	1
miR-377	1	30	20	3.34	1
miR-26a-5p	1	49	6	3.10	1
miR-214	1	30	20	2.90	1
miR-150-5p	1	49	6	2.70	1
miR-370	1	30	20	2.68	1
let-7f-5p	1	49	6	2.60	1
miR-26b-5p	1	49	6	2.60	1
let-7c-5p	1	49	6	2.50	1
miR-23b-3p	1	49	6	2.40	1
miR-125b-5p	1	49	6	2.30	1
miR-126-3p	1	49	6	2.30	1
miR-195-5p	1	49	6	2.20	1
miR-424-5p	1	49	6	2.20	1
miR-374a-5p	1	49	6	2.10	1
let-7a-5p	1	49	6	2.00	1
let-7e-5p	1	49	6	2.00	1
miR-125a-5p	1	49	6	2.00	1
miR-542-5p	1	14	10	2.00	1
miR-337-5p	1	30	20	1.94	1
miR-1305	1	73	31	1.77	1
miR-758	1	30	20	1.61	1
miR-300	1	30	20	1.56	1
miR-93	1	176	100	NR	0
miR-125	1	67	15	NR	0
miR-34	1	67	15	NR	0
miR-30a-3p	1	40	26	NR	0
miR-301b	1	34	14	NR	0
miR-10b	1	28	14	NR	0
miR-195	1	28	14	NR	0
miR-30a-5p	1	28	14	NR	0
miR-101	1	21	7	NR	0
miR-10b^*^	1	21	7	NR	0
miR-133a	1	21	7	NR	0
miR-133b	1	21	7	NR	0
miR-152	1	21	7	NR	1
miR-29b	1	21	7	NR	0
miR-34b	1	21	7	NR	0
miR-411	1	21	7	NR	0

NR = not reported, ^**^ mean fold change as found by qRT-PCR.

For 7 miRNAs (miR-203, miR-21, miR-204, miR-9, miR-199b, miR-99a and miR-100) an inconsistent altered expression was found (Table 3). MiR-203 was found up-regulated in 3 studies with mean fold change 4.19, however was also found down-regulated in one study [23, 35, 40, 41].

**Table 3:** MiRNAs with inconsistent direction of change

MiRNA	Number of studies	Up/down-regulation	Sample size EC	Sample size control	Mean fold change^**^	Studies with fold change reported
miR-203	3	Up	111	59	4.19	2
	1	Down	60	10	0.073	1
miR-21	1	Up	67	15	NR	0
	1	Down	40	26	NR	0
miR-204	1	Up	26	22	5.79	1
	1	Down	46	28	NR	0
miR-9	1	Up	73	31	5.46	1
	1	Down	34	14	NR	0
miR-199b	1	Up	4	14	2.89	1
	1	Down	73	31	3.52	1
miR-99a	1	Up	4	14	1.96	1
	1	Down	73	31	3.29	1
miR-100	1	Up	4	14	1.65	1
	1	Down	73	31	2.56	1

NR = not reported, ^**^mean fold change as found by qRT-PCR.

### Differentially expressed miRNAs per specimen type

The included articles were subdivided according to specimen subtype: tissue specimens (19 articles), serum/plasma (8 articles), urine (1 article) and LBC (1 article) (Supplementary Tables 2–9). For tissue specimens, the results were similar to the previously reported results with the only difference being that miR-200c was listed in 7 articles (previously 8) and miR-223 in 2 (previously 5). For serum/plasma samples miR-223 was most often reported up-regulated in 3 studies, followed by miR-222, miR-186 and miR-203 in 2 studies. MiR-205 was not reported in serum/plasma samples to be deregulated.

### Differentially expressed miRNAs per subtype EC

Furthermore, the articles were subdivided according to EC subtype: looking at endometrioid (type I) versus serous (type II) only, to distinguish if a miRNA signature can be found per subtype. There were 12 studies looking at endometrioid carcinoma only [24, 25, 35–38, 40, 42–45] and one study by Devor et al. who reported on both endometrioid and serous carcinomas but reported the subtypes separately [33]. There was only one other study looking at serous carcinoma only [32]. For endometrioid type tumours, data was in line with previously reported data; the most often up-regulated miRNA was miR-205 (cited in 7 articles), followed by miR-200c (6 articles), miR-182, and -200a (5 articles), miR-183 (4 articles) and miR-135b, -429, -141 and -223 (3 articles), data not shown. For serous type endometrial carcinoma, miR-205 was found up-regulated in both studies, furthermore miR-200c, -135a and -135b were up-regulated in one of the two studies.

## DISCUSSION

Endometrial cancer is the most common malignancy of the female genital tract in developed countries with rising incidence and mortality rates [46]. Although EC is generally associated with good prognosis, patients presenting with advanced or recurrent EC have poor survival rates [6]. MiRNAs have been shown to play a significant role in tumour genesis and progression and therefore warrant a clinical potential as diagnostic and prognostic marker in EC. In this review a systematic search was conducted to identify the feasibility and overall diagnostic value of miRNA expression in EC.

MiR-205 was most consistently found to be up-regulated, with a differential expression reported among ten studies and mean fold change of 198.08 [28, 31–33, 35, 36, 40, 42, 44, 45]. MiR-205 is involved in the regulation of PTEN expression in endometrial cancer and leads to reduced cell apoptosis [47]. Furthermore, miR-205 represses the tumour suppressor gene JPH4, promoting tumorigenesis and tumour progression [47]. However, miR-205 is not only up-regulated in endometrial cancer, but also in other cancer sites such as lung and ovarian cancer [48, 49]. Therefore, miR-205 on its own seems not adequate as a diagnostic test for the detection of endometrial cancer. Lee et al. found a panel of six miRNAs (miR-205, miR-200a, miR-200c, miR-182, miR-183 and miR-21) to have an area under the curve (AUC) of 0.961, sensitivity and specificity of 91% and 94% respectively in discriminating endometrial cancers from hyperplasia or normal tissue [45]. The results of this systematic review confirm the importance of these miRNAs in endometrial carcinogenesis.

MiR-200c and miR-200a were reported consistently up-regulated in 8 and 5 studies respectively, in addition miR-200b, miR-429 and miR-141 were reported up-regulated in 3 studies. These miRNAs are part of the miR-200 family, the expression and function of which has been well documented in various tissues and has been suggested to play an important role in inhibiting cell malignant transformation and preventing tumour initiation [50]. The miR-200 family targets the expression of many genes, including ZEBs (Zinc finger E-box-binding homeobox), which are the transcription factors that regulate cellular transformation, more specifically epithelial-to-mesenchymal transition (EMT), during cancer development and progression through repression of adhesion molecules such as E-cadherin [51]. Members of the miR-200 family also host diagnostic and prognostic potential in other cancer sites such as gastric, ovarian, lung and colorectal cancer [52–55].

Furthermore, miR-182, miR-183 and miR-223 were found up-regulated in 5 articles and miR-135b was found to be up-regulated in 3 articles. MiR-182 promotes cell proliferation by targeting the tumour suppressor gene TCEAL7, miR-183 targets CPEB1 while miR-223 targets IGF-1R [56–58]. MiR-135b targets FOXO1 to promote cell proliferation in EC cells [59]. These miRNAs are also up-regulated in patients with non-small cell lung cancer, colorectal, prostate and pancreatic cancer [60–62]. In addition, a recent systematic review by Delangle et al. found miR-182 and miR-183 to be associated with poorer prognosis in terms of overall survival and recurrence-free survival in endometrial cancer [63]. They therefore conclude that miRNA analysis merits a role as a prognostic factor in the management of patients with EC. For other gynaecological cancer sites such as ovarian and cervical cancer the diagnostic and prognostic significance of different panels of miRNAs have been investigated and also show promising results [19, 64].

The distinct panel identified in this systematic review (miR-205, the miR-200 family, miR-135b, -182, -183, and -223) is promising in the detection of endometrial cancer. However, some of the same miRNAs are also upregulated in colorectal cancer, therefore, we suggest that these miRNAs may be used in the diagnosis of women presenting with specific symptoms such as abnormal or postmenopausal bleeding.

Since miRNAs can be detected in a huge variety of bodily fluids including urine and since miRNAs are stable in urine, urine seems like a promising non-invasive test for the detection of EC [65]. Urinary miRNAs have shown potential in the detection of bladder and prostate cancer [66, 67], however in EC only one study has used urine for the detection of miRNA [26]. In addition, Zavesky et al. compared urinary miRNA expression levels of pre- and post-surgery ovarian cancer samples and between patients with ovarian and endometrial cancer (*n* = 10) and healthy controls and proposed urinary miRNA should be further investigated to test the diagnostic potential of urine miRNAs in gynaecological cancers [68]. A urinary diagnostic test will potentially allow for easier access to care, help reduce anxiety among women and could prevent the need for painful biopsies. Another potential is possibly reducing the burden of travelling long distances to the hospital and costs for patients. In addition, if fewer patients need to be referred to the hospitals this will have a potential cost reduction implication. Further studies should therefore determine if urinary miRNA detection is a valid non-invasive way of reliably detecting EC.

In addition to the need for new biomarkers to detect endometrial carcinoma, there is also the need for these biomarkers to accurately distinguish between low (grade 1 and grade 2) or high grade (grade 3) endometrioid endometrial carcinoma (EEC) [69]. Ratner et al. reported unique miRNA signatures for endometrial type I endometrioid carcinomas, type II papillary serous carcinomas and uterine carcinomas, but no difference between grade 1 and grade 3 endometrioid tumours [70]. Further research may help determine if miRNA can accurately distinguish low grade EEC from high grade EEC.

Furthermore, there is an increasing interest in improving the preoperative classification of EC, in order to allow for non-invasive and more precise diagnostic options for patients. In 2013, the Cancer Genome Atlas proposed an additional division of EC into four molecular subtypes: Polymerase-ε (POLE) ultramutated, microsatellite instability (MSI) hypermutated, copy-number (CN) low and CN high [71]. CN low include most endometrioid tumours and are frequently associated with mutations in PTEN, CTNNB1, PIK3CA, ARID1A and KRAS, whereas CN high include serous tumours and 25% of high grade endometroid tumours [72]. POLE and MSI mutated EC tumours show better survival outcomes [71]. Since miR-205 is involved in the regulation of PTEN expression in endometrial cancer, miRNA detection could potentially be of use in a molecular based classification system for correct preoperative diagnosis and classification of EC.

Although these findings are encouraging, the main limitations to the usage of miRNAs include different platforms of miRNA profiling, including microarray, next generation sequencing and RT-qPCR, which leads to inconsistency and difficulties in comparing results [73]. It is difficult to compare data gained with different miRNA profiling platforms, as they are only somewhat reproducible and even intraplatform variation is common [74]. Due to differences in the accuracy, reproducibility, sensitivity and specificity of PCR kits, the reproducibility of miRNA detection and quantification is relatively low [75]. Furthermore, most miRNAs are not cancer type specific, therefore an EC specific miRNA signature needs further testing to determine if these miRNAs can accurately distinguish EC from benign tissue. This systematic review has shown the most promising miRNAs to be miR-205, -200a, -200b, -200c, -141, -429, -135b, -182, -183 and -223 and therefore need further testing.

The strengths of this study include a comprehensive systematic search performed by two reviewers independently. In addition, to improve comparability, we have only included studies in this review that used RT-qPCR for miRNA detection. Furthermore, all included studies varied in the subtype EC which they assessed; some studies included only type I (endometrioid tumours), others only type II (all other tumours) and some studies combined the two subtypes in their analyses, even though type I and type II vary in pathophysiology and prognosis. However, when subdividing the articles into endometrioid versus serous endometrial carcinoma, the data found seems in line with data found when combining subtypes.

The limitations to this systematic review are the following; there is a high heterogeneity in methodologies used (different platforms, analysis software and normalisation strategies) and specimen samples (FFPE, fresh frozen tissue, serum, plasma, urine, LBC) among the different studies included. When subdividing the articles per specimen type, we found a different expression for tissue specimens compared to serum/plasma samples. In addition, the majority of studies only included small sample sizes. Finally, the majority of studies were conducted in Asia and no studies were conducted in Africa, South-America or Oceania.

In conclusion, this systematic review shows that miRNAs are potential promising biomarkers for the diagnosis of EC, however no sufficient high-quality evidence is available to draw hard conclusions. The combination of miR-205, the miR-200 family, miR-135b, -182, -183 and -223 needs further testing in larger studies with standardised protocols to improve the accuracy of using these miRNAs in diagnosing EC in the future. In addition, the potential of urine as a non-invasive biofluid should be investigated.

### Clinical significance

MiRNA can potentially be used in low resource settings where there is lack of trained histopathologists. In addition, a urinary miRNA test can potentially be used as a non-invasive test for the detection of EC. This will allow for easier access to care and reduction of travelling long distances to the hospital for patients. It could mean a cost reduction for the hospital, if patients can be seen in the community instead of hospitals.

## MATERIALS AND METHODS

### Search strategy

This review was performed according to Preferred Reporting Items for Systematic reviews and Meta-Analyses (PRISMA) guidelines, and in accordance with the principles outlined in the Cochrane Handbook [76]. Systematic searches were performed in Medline (1946 until May 2019), Embase (1980 until May 2019) and Cinahl (1981 until May 2019) and the Cochrane Controlled Register of Trials with the following terms: (‘microRNA’ OR ‘miRNA’ OR ‘miR’) AND (‘endometrial cancer’ OR ‘endometrial carcinoma’ OR ‘endometrial neoplasm’ OR ‘uter^*^ carcinoma’ OR ‘uter^*^ cancer’ OR ‘uter^*^ neoplasm’). Search strategies were adapted accordingly to each database. In addition, grey literature was searched including abstracts of scientific meetings as well as manually checking the reference lists of eligible studies to identify any additional studies to include in this review.

### Study inclusion/exclusion criteria

Studies were considered eligible if publications met all the following criteria: (1) the study concerned the diagnostic value of miRNAs; (2) histological subtype was specified as primary endometrial cancer; (3) studies used real-time quantitative reverse transcription polymerase chain reaction (qRT-PCR) techniques to detect miRNA expression, (4) the study was in English; (5) the study was conducted in human subjects; (6) the study was not a review, abstract or editorial article. Cell line models were excluded due to the limitations cell lines models have in terms of the *in vitro* adaptation of cells to culture conditions, which sometimes leads to the discrepancy between the experimental and clinical outcomes [77].

### Study selection

Two reviewers (HD and KG) independently assessed titles and abstracts of all identified studies. Those studies that clearly did not meet the inclusion criteria were excluded. Potentially relevant studies were retrieved in full text and were further reviewed for eligibility by both reviewers.

### Data extraction

Data extraction was completed by two reviewers (HD and KG) and disagreements were resolved by consensus. The necessary information and data were extracted from the final eligible articles as follows: first author, year of publication, country of origin, number of cases and controls, histology type, miRNA expression test methods, type of specimens, cut-off values, expression changes and sensitivity and specificity.

### Trial quality assessment

The methodological qualities of the selected eligible articles were assessed by the Quality Assessment of Diagnostic Accuracy Studies 2 (QUADAS-2) score system [78]. The QUADAS-2 tool combines the patient selection index, index test, reference standard, flow and timing to evaluate risk of bias and applicability. The seven items (four items on bias risk and three items on applicability) were assessed for all included articles. Two authors independently tested the pilot QUADAS-2 items (HD and KG) and discrepancies were resolved by consensus.

### Ranking of miRNA

In order to collect and sum up the results of the included studies, the vote-counting strategy was used [79]. The vote-counting strategy ranks biomarkers on the basis of one principal and two secondary criteria and is the most common and most frequently cited strategy to rank biomarker candidates systematically [79, 80]. The principal criterion is made up of the number of studies in which each study showing significant differential expression in the same direction (either up- or down-regulated) for a biomarker counts as a vote in favour of that biomarker being real. The secondary criteria are (1) total sample size summed across all of the supporting studies (the assumption being that larger studies tend to be more reliable) and (2) mean fold change (based on the idea that large differences in biomarker expression are more likely to be confirmed than small differences).

## SUPPLEMENTARY MATERIALS





## Abbreviations

AUC: area under the curve
EC: endometrial cancer
EEC: endometrioid endometrial carcinoma
EMT: epithelial-to-mesenchymal transition
FFPE: Formalin-Fixed, Paraffin-Embedded
LBC: liquid based cytology
MiRNAs: microRNAs
qRT-PCR: real-time quantitative reverse transcription polymerase chain reaction
QUADAS-2: Quality Assessment of Diagnostic Accuracy Studies 2
UK: United Kingdom
ZEBs: Zinc finger E-box-binding homeobox
