# Supplementary material for: Diagnostic value of microRNA panel in endometrial cancer: A systematic review
Source: Oncotarget. 2020 May 26;11(21):2010–23. doi: 10.18632/oncotarget.27601 (PMC7260115; doi:10.18632/oncotarget.27601)
Supplement: Supplementary file 1 [file oncotarget-11-2010-s001.pdf]

Diagnostic value of microRNA panel in endometrial cancer: A systematic review

SUPPLEMENTARY MATERIALS

Supplementary Table 1: The main characteristics of included studies. See Supplementary Table 1

Supplementary Table 2: Up-regulated miRNAs in tissue samples

MiRNA	Number of studies	Sample size EC	Sample size control	Mean fold change**	Studies with fold change reported
miR-205	10	134	63	198.08	4
miR-200c	7	126	55	27.99	4
miR-182	5	141	79	11.41	3
miR-183	5	141	79	8.75	2
miR-200a	5	121	75	5.20	4
miR-135b	3	96	35	35.59	2
miR-429	3	117	61	6.83	3
miR-200b	3	103	51	3.86	2
miR-200a*	2	73	31	26.96	1
miR-141-3p	2	65	30	22.95	2
miR-200b*	2	73	31	6.52	1
miR-223	2	38	28	4.9	1
miR-141	2	30	20	4.70	1
miR-106a	2	68	48	2.80	2
miR-135a	1	23	4	34.05	1
miR-205-5p	1	16	24	31.52	1
miR-200b-3p	1	16	24	27.68	1
miR-182-5p	1	16	24	27.30	1
miR-200c-3p	1	16	24	24.40	1
miR-92a	1	39	17	15.63	1
miR-9-5p	1	16	34	15.05	1
miR-9	1	73	31	5.46	1
miR-210	1	38	28	5.23	1
miR-96	1	73	31	4.27	1
miR-194	1	38	28	4.11	1
miR-95	1	38	28	4.09	1
miR-155	1	38	28	3.87	1
miR-18a*	1	30	20	3.65	1
miR-96-5p	1	49	6	3.20	1
miR-103	1	38	28	3.00	1
miR-151	1	38	28	2.85	1
miR-34a	1	30	20	2.63	1
miR-92a-1*	1	30	20	2.47	1
miR-20a*	1	30	20	2.34	1
miR-106b*	1	30	20	2.34	1
miR-17*	1	30	20	1.99	1
miR-185	1	30	20	1.85	1
miR-222-3p	1	16	24	1.80	1
miR-146	1	141	20	NR	0
miR-425	1	141	20	NR	0
miR-944	1	68	20	NR	0
miR-15b	1	67	15	NR	0
miR-16	1	67	15	NR	0
miR-128a	1	67	15	NR	0
miR-148b	1	67	15	NR	0
miR-196a	1	67	15	NR	0
miR-301	1	67	15	NR	0
miR-582-5p	1	67	15	NR	0
miR-499	1	28	14	NR	0

NR = not reported, **mean fold change as found by qRT-PCR.

Supplementary Table 3: Up-regulated miRNAs in serum/plasma samples

MiRNA	Number of studies	Sample size EC	Sample size control	Mean fold change**	Studies with fold change reported
miR-223	3	66	71	57.81	2
miR-222	2	26	22	19.16	1
miR-186	2	26	22	11.39	1
miR-203	2	79	44	NR	0
miR-15b	1	40	49	6.10	1
miR-204	1	26	22	5.79	1
miR-27a	1	40	49	5.63	1
miR-199b	1	34	14	2.89	1
miR-887-5p	1	50	50	2.41	1
miR-99a	1	34	14	1.96	1
miR-100	1	34	14	1.65	1
miR-200a	1	34	14	NR	0
miR-141	1	34	14	NR	0
miR-92a	1	34	14	NR	0
miR-449a	1	34	14	NR	0
miR-1228	1	34	14	NR	0
miR-1290	1	34	14	NR	0
miR-205	1	12	12	NR	0
miR-135b	1	12	12	NR	0

NR = not reported, **mean fold change as found by qRT-PCR.

Supplementary Table 4: Up-regulated miRNAs in urine samples

MiRNA	Number of studies	Sample size EC	Sample size control	Mean fold change**	Studies with fold change reported
miR-200c-3p	1	22	5	NR	0

NR = not reported, **mean fold change as found by qRT-PCR.

Supplementary Table 5: Up-regulated miRNAs in LBC samples

MiRNA	Number of studies	Sample size EC	Sample size control	Mean fold change**	Studies with fold change reported
miR-182-5p	1	12	28	18.21	1
miR-205-5p	1	12	28	16.85	1
miR-141-3p	1	12	28	11.45	1
miR-200c-3p	1	12	28	6.27	1
miR-200b-3p	1	12	28	4.69	1
miR-222-3p	1	12	28	3.43	1

NR = not reported, **mean fold change as found by qRT-PCR.

Supplementary Table 6: Down-regulated miRNAs in tissue samples

MiRNA	Number of studies	Sample size EC	Sample size control	Mean fold change**	Studies with fold change reported
miR-137	1	23	4	115.15	1
miR-129-3p	1	23	4	42.30	1
miR-410	1	73	31	13.91	1
miR-503	1	14	10	8.60	1
miR-1247	1	30	20	5.31	1
miR-376c	1	30	20	3.64	1
miR-199b	1	73	31	3.52	1
miR-377	1	30	20	3.34	1
miR-99a	1	73	31	3.29	1
miR-26a-5p	1	49	6	3.10	1
miR-214	1	30	20	2.90	1
miR-150-5p	1	49	6	2.70	1
miR-370	1	30	20	2.68	1
let-7f-5p	1	49	6	2.60	1
miR-26b-5p	1	49	6	2.60	1
miR-100	1	73	31	2.56	1
let-7c-5p	1	49	6	2.50	1
miR-23b-3p	1	49	6	2.40	1
miR-125b-5p	1	49	6	2.30	1
miR-126-3p	1	49	6	2.30	1
miR-195-5p	1	49	6	2.20	1
miR-424-5p	1	49	6	2.20	1
miR-374a-5p	1	49	6	2.10	1
let-7a-5p	1	49	6	2.00	1
let-7e-5p	1	49	6	2.00	1
miR-125a-5p	1	49	6	2.00	1
miR-542-5p	1	14	10	2.00	1
miR-337-5p	1	30	20	1.94	1
miR-1305	1	73	31	1.77	1
miR-758	1	30	20	1.61	1
miR-300	1	30	20	1.56	1
miR-125	1	67	15	NR	0
miR-34	1	67	15	NR	0
miR-30a-3p	1	28	14	NR	0
miR-10b	1	28	14	NR	0
miR-195	1	28	14	NR	0
miR-30a-5p	1	28	14	NR	0
miR-101	1	21	7	NR	0
miR-10b*	1	21	7	NR	0
miR-133a	1	21	7	NR	0
miR-133b	1	21	7	NR	0
miR-152	1	21	7	NR	1
miR-29b	1	21	7	NR	0
miR-34b	1	21	7	NR	0
miR-411	1	21	7	NR	0

NR = not reported, **mean fold change as found by qRT-PCR.

Supplementary Table 7: Down-regulated miRNAs in serum/plasma samples

MiRNA	Number of studies	Sample size EC	Sample size control	Mean fold change**	Studies with fold change reported
miR-93	1	176	100	NR	0
miR-204	1	46	28	NR	0
miR-30a-3p	1	40	26	NR	0
miR-9	1	34	14	NR	0
miR-301b	1	34	14	NR	0
miR-21	1	12	12	NR	0

NR = not reported, **mean fold change as found by qRT-PCR.

Supplementary Table 8: MiRNAs with inconsistent direction of change in tissue

MiRNA	Number of studies	Up/down-regulation	Sample size EC	Sample size control	Mean fold change**	Studies with fold change reported
miR-203	2	Up	111	59	4.19	2
	1	Down	60	10	0.073	1
miR-21	1	Up	67	15	NR	0
	1	Down	28	14	NR	0

NR = not reported, **mean fold change as found by qRT-PCR.

Supplementary Table 9: MiRNAs with inconsistent direction of change in plasma/serum

MiRNA	Number of studies	Up/down-regulation	Sample size EC	Sample size control	Mean fold change**	Studies with fold change reported
MiR-204	1	Up	26	22	5.79	1
	1	Down	46	28	NR	0

NR = not reported, **mean fold change as found by qRT-PCR.
