# Supplementary material for: Diagnostic value of microRNA panel in endometrial cancer: A systematic review
Source: Oncotarget. 2020 May 26;11(21):2010–23. doi: 10.18632/oncotarget.27601 (PMC7260115; doi:10.18632/oncotarget.27601)
Supplement: Supplementary file 2 [file oncotarget-11-2010-s002.docx]

**Supplementary Table 1: The main characteristics of included studies**

| **Author** | **Country** | **Subtype** | **Sample size**  *Cancer Hyperplasia Controls* | | | **Specimen** | **Test method** | **Outcome**** | **Sensitivity / specificity** |
| --- | --- | --- | --- | --- | --- | --- | --- | --- | --- |
| Hiroki (2010) | Japan | Serous | 21 | - | 7 | FFPE | Microarray and RT-qPCR | Up-regulated:  miR-205  Down-regulated:  miR-101, miR-10b*, miR-133a, miR-133b, miR-152, miR-29b, miR-34b, miR-411 | - |
| Jayaraman (2017) | USA | Not specified | 49 | - | 6 | FFPE | RT-qPCR | Up-regulated:  miR-141-3p, miR-96-5p  Down-regulated:  miR-26a-5p, miR-150-5p, let-7f-5p, miR-26b-5p, let-7c-5p, miR-125a-5p, miR-195-5p, miR-23b-3p, miR-374a-5p, miR-126-3p, miR125b-5p, miR-424-5p, let-7-a-5p, let-7e-5p | - |
| Torres (2012) | Poland | Endometrioid | **Fresh frozen**  30  **FFPE**  43  **Plasma**  34 | **Fresh frozen**  -  **FFPE**  -  **Plasma**  - | **Fresh frozen**  16  **FFPE**  15  **Plasma**  14 | Fresh frozen, FFPE and plasma | Microarray and RT-qPCR | **Tissue:**  Up-regulated:  miR-9, miR-92a, miR-96, miR-135b, miR-182, miR-183, miR-200a, miR-200a*, miR-200b, miR-200b*, miR-200c, miR-203, miR-205, miR-429  Down-regulated:  miR-410, miR-1305  **Plasma:**  Up-regulated:  miR-92a, miR-141, miR-200a, miR-203, miR-449a, miR-1228, miR-1290  Down-regulated:  miR-9, miR-301b | **Tissue:**  miRNA signature miR-92a/miR-410 AUC 0.977 and miR-92a/miR-205/miR-410 AUC 0.984.  **Plasma:**  miRNA signature miR-9/miR-1228 AUC 0.909 and miR-9/miR-92a AUC 0.913 |
| Karaayvaz (2012) | USA | All subtypes | 48 | - | 5 | FFPE | Microarray and RT-qPCR | Up-regulated:  miR-200c, miR-205 | - |
| Tsukamoto (2015) | Japan | Endometrioid | **Fresh tissue**  28  **Plasma**  12 | **Fresh tissue**  -  **Plasma**  - | **Fresh tissue**  14  **Plasma**  12 | Fresh tissue samples and plasma | Next-generation sequencing and RT-qPCR | **Tissue:**  Up-regulated:  miR-499, miR-135b, miR-205  Down-regulated:  miR-10b, miR-195, miR-30a-5p, miR-30a-3p, miR-21  **Plasma:**  Up-regulated:  miR-135b, miR-205  Down-regulated:  mi-30a-3p and miR-21 | **Tissue:** miRNA signature miR-135b/miR-195 AUC 0.9835 and miR-135b/mi-R30a-3p AUC 0.9898  **Plasma:**  AUC for miR-21: 0.7569,  AUC for miR-30-a-3p: 0.8125  AUC for miR-135b: 0.9722 AUC for miR-205: 1.0 |
| Wilczynski (2018) | Poland | Endometrioid | 90 | - | 10 | FFPE | RT-qPCR | Up-regulated:  miR-200c | - |
| Fang (2018) | China | Not specified | 176 | - | 100 | Serum | RT-qPCR | Down-regulated:  miR-93 | AUC: 0.781 (0.724-0.842) |
| Kottaridi (2017) | Greece | Not specified | **FFPE**  16  **LBC**  12 | **FFPE**  8  **LBC**  6 | **FFPE** 34  **LBC**  28 | FFPE and liquid based cytology (LBC) | RT-qPCR | **FFPE:**  Up-regulated:  miR-9-5p, miR141-3p, miR182-5p, miR-200b-3p, miR-200c-3p, miR205-5p  **LBC:**  Up-regulated:  miR-182-5p, miR-141-3p, miR-200b-3p, miR-200c-3p, miR-205-5p, miR-222-3p | **FFPE:** miR-182-5q ROC 0.979, 4-fold overexpression sensitivity 93.8%, specificity over 95%  miR-141-3p and 13-fold increase AUC 0.964, sensitivity 81.3%  miR-205-5p 8 fold increase AUC 0.964, sensitivity 81.3%  **LBC** similar results with lower AUCS; miR-141-3p 0.955, miR-182-5 0.942 with sensitivity of 85.7% and specificity of 100% for 5-fold increase. |
| He (2017) | China | All types | 68 | - | 20 | Liquid nitrogen and FFPE | RT-qPCR | Up-regulated:  miR-944 | - |
| Jiang (2016) | China | Not specified | 50 | - | 50 | Serum | RT-qPCR | Up-regulated:  miR-887-5p | AUC for EC diagnosis 0.728, specificity 0.60, sensitivity 0.95 (0.563-0.892) |
| Benati (2017) | Italy | Clear cell, endometrioid and serous | 45 | - | 30 | Serum | RT-qPCR | Up-regulated:  miR-203 | AUC 0.71 |
| Srivastava (2018) | USA | Not specified | 22 | - | 5 | Urine | RT-qPCR | Up-regulated:  miR-200c-3p | - |
| Lu (2016) | China | All types | 67 | - | 15 | Snap-frozen samples | Microarray and RT-qPCR | Up-regulated:  miR-21, miR-196a, miR-16, miR-582-5p, miR-15b, miR-301, miR-148b, miR-128a  Down-regulated:  miR-125, miR-34 | - |
| Wang (2014) | China | Endometrioid | 40 | 4 | 49 | Plasma | RT-qPCR | Up-regulated:  miR-27a, miR-15b, miR-223 | AUCs: 0.768 for miR-15b; 0.813 for miR-27a; 0.768 for miR-223.  Combining miR-27a and CA125: AUC 0.894 |
| Lee (2013) | Korea | Endometrioid | **FFPE**  20  **Fresh frozen**  4 | **FFPE**  -  **Fresh frozen**  - | **FFPE**  10  **Fresh frozen**  **4** | FFPE and fresh frozen tissue | Microarray and RT-qPCR | Up-regulated:  miR-200a*, miR-205, miR-141, miR-200b*, miR-182 | - |
| Wilczynski (2016) | Poland | Endometrioid | 90 | - | 10 | FFPE | RT-qPCR | Up-regulated:  miR-205 | - |
| Jia (2013) | China | Endometrioid | 26 | - | 22 | Serum | RT-qPCR | Up-regulated:  miR-222, miR-223, miR-186, miR-204 | ROC curve of four-serum miRNA signature was 0.927 (individual ones ranging from 0.727 to 0.837).  AUC of 0.927, specificity 87.5% and sensitivity 91.7% |
| Cohn (2010) | USA | All types | 141 | - | 20 | FFPE | Microarray and RT-qPCR | Up-regulated:  miR-200c, miR-183, miR-205, miR-223 and miR-425 | Cluster sensitivity 92.% and positive predictive value 97% |
| Lee (2012) | Korea | Endometrioid | 22 | 43 | 10 | FFPE | RT-qPCR | Up-regulated:  miR-182, miR-183, miR-200a, miR-200c, miR-205 | Composite panel of six miRNAs sensitivity 91% and specificity 94%  Individual 64-77% sensitivity and 66-91% specificity. |
| Snowdon (2011) | Canada | Endometrioid | 14 | 10 | 10 | FFPE | Microarray and RT-qPCR | Up-regulated:  miR-200a, miR-429  Down-regulated:  miR-503, miR-542-5p | - |
| Montagnana (2017) | Italy | Not specified | 46 | - | 28 | Serum | RT-qPCR | Up-regulated:  miR-186, miR-222, miR-223  Down-regulated:  miR-204 | - |
| Jurcevic (2016) | Sweden | Endometrioid | 30 | - | 20 | FFPE | RT-qPCR | Up-regulated:  miR-183, miR-182, miR-429, miR-200b, miR-200a, miR-141, miR-18a, miR-200c, ,miR-18a*, miR-106a, miR-17, miR-34a, miR-92a-1*, miR-106b*, miR-20a*, miR-17*, miR-185  Down-regulated:  miR-1247, miR-376c, miR-377, miR-370, miR-214, miR-337-5p, miR-300, miR-758 | - |
| Chung (2009) | Hong Kong | Endometrioid | 38 | - | 28 | Snap-frozen tissue | RT-qPCR | Up-regulated:  miR-205, miR-182, miR-200a, miR-223, miR-210, miR-200c, miR-183, miR-155, miR-203, miR-194, miR-95, miR-106a, miR-103, miR-151 | - |
| Al-Deresawi (2018) | Iraq | Endometrioid | 60 | - | 10 | Tissue specimen | RT-qPCR | Down-regulated:  miR-203 | - |
| Devor (2017) | USA | Endometrioid and serous | 23 | - | 4 | Snap-frozen tissue | RT-qPCR | Up-regulated:  miR-135a, miR-135b, miR-200c, miR-205  Down-regulated:  miR-137, miR-129-3p | - |
| Torres (2012) | Poland | Endometrioid | **Tissue:**  73  **Plasma:**  4 | - | **Tissue:**  31  **Plasma:**  14 | Fresh frozen tissue and FFPE | RT-qPCR | **Tissue:**  Down-regulated:  miR-99a, miR-100, miR-199b  **Plasma:**  Up-regulated:  miR-99a, miR-100, miR-199b | AUC miR-199b tissue 0.704.  AUC miR-99a plasma 0.810.  2-miRNA signature based on miR-99a and miR-199b in plasma sensitivity 88% and specificity 93% |
| ** MiRNAs only reported in table if detected or confirmed with PCR. | | | | | | | | | |
